# Patient Blood Management in Hematology: Focusing on Platelet Transfusion

**DOI:** 10.3390/jcm14238434

**Published:** 2025-11-27

**Authors:** Pilar Solves, Micaela Córcoles, Montiel Soriano, Brais Lamas, David Martínez-Campuzano, Pedro Asensi, Inés Gómez-Seguí, Javier de la Rubia

**Affiliations:** 1 Haematology Department, Hospital Universitari i Politècnic La Fe, 46026 Valencia, Spain; corcoles_mic@gva.es (M.C.); gomez_ine@gva.es (I.G.-S.);; 2 CIBERONC, Instituto Carlos III, 28029 Madrid, Spain; 3 School of Medicine and Dentistry, Catholic University of Valencia, 46001 Valencia, Spain

**Keywords:** platelet transfusion, patient blood management, transfusion audit

## Abstract

**Background**: Platelet transfusion can be life-saving in some situations, but, if unnecessary, it puts the patients at risk and increases health system costs. Previous reports of audits performed on platelet transfusion practice in hospitals from different countries show a low adherence of physicians to the current recommendation. The objective of this article is to describe our patient blood management strategy to improve platelet transfusion practice of hematologic inpatients and outpatients, focusing on the impact on platelet transfusion activity and appropriateness rate. **Methods**: We developed a specific platelet transfusion guideline for hematological patients at our hospital. Platelet transfusion activity was audited after implementing the guide in mid-2024 and mid-2025, and these data were compared to the previous audits performed before the intervention. We also reviewed the platelet transfusion activity and adverse effects related to from 2021 to 2024. **Results**: Audit 1 reviewed 128 inpatients and 49 outpatients transfused from March 2022 to September 2023. Audit 2 studied 130 inpatients and 45 outpatients requiring platelet transfusion from March 2025 to July 2025. Annual platelet transfusions decreased from 8028 before intervention to 6880 in 2024 (14% decrease). In the hematological area, where the intervention has been made, the reduction in platelet transfusions was of 15.6% in hospitalized patients and 34.5% in outpatients as compared to 2023 (*p* < 0.001). The platelet supply decreased from 8407 in 2023 to 7138 in 2024. Adverse events related to PLT transfusion occurred in 51 patients in 2023 and 35 patients in 2024. **Conclusions**: Implementing patient blood management strategies focused on platelets led to a more rational and standardized use of platelet transfusions and decreased transfusion burden. Each organization should establish thresholds for platelet transfusion based on available evidence and guidelines, and also audit adherence to guidelines. In view of the scarce available evidence, research on platelet transfusion practice is mandatory.

## 1. Introduction

Platelet (PLT) transfusion is a supportive treatment widely used to prevent (prophylactic) and treat active bleeding in patients with thrombocytopenia [1]. Annually, more than 240.000 platelet concentrates are transfused in Spain [2] and approximately two million platelet components are transfused in the United States [3]. While demand for red blood cell transfusions has moderated, the platelet request continues to rise. In 2024, the platelet transfusion episodes increased a 10% as compared to 2021 in Spanish hospitals [2]. More than 50% of platelets are transfused to hematological patients affected by malignant and non-malignant conditions for prophylactic purposes [4].

Platelet transfusion practice is based on available guidelines that mostly include moderate–low evidence-based indications [5,6]. The available strongest evidence supports the prophylactic transfusion strategy using low-dose platelets and a threshold of 10,000/µL for hematological patients undergoing chemotherapy or allogeneic stem cell transplantation. This threshold should be increased to 20,000/µL if there are some additional-risk factors for bleeding [7]. The lowest evidence lies in indications for pre-procedure platelet transfusions. For instance, there are differences among guidelines for lumbar puncture. While the British guidelines recommend a platelet threshold of <40,000/µL [5], the most recent AABB guidelines establish that platelet transfusion should be administered when the platelet count is <20,000/µL [6], taking into account the low event rate estimate for this procedure.

PLT transfusion can be life-saving in some situations, but if unnecessary, it puts the patients at risk and increases health system costs. However, previous reports of audits performed on PLT transfusion practice in hospitals from different countries show a low adherence of physicians to the current recommendations [8]. In fact, audits show that more than 40% of platelet transfusion episodes are considered inappropriate [9,10,11].

Platelets are a limited and expensive resource whose availability is not always guaranteed. On the contrary, a shortage can occur at times and compromise the treatment of patients in need [12]. Thus, platelet transfusion practice in hematological patients is clearly an area for improvement. In this line, implementing patient blood management (PBM) strategies in hematological units can have a high impact on blood component consumption and patient safety. Use of blood components according to the available guidelines based on evidence is a key strategy of PBM programs [13]. While PBM programs are usually focused on red blood cell transfusion, we consider that in hematological patients, PBM strategies should be primarily applied to platelet transfusion practice.

The objective of this article is to describe our PBM strategy in the platelet transfusion practice of hematologic inpatients and outpatients, focusing on the impact on platelet transfusion activity and appropriateness rate.

## 2. Methods

### 2.1. Evaluation of the Platelet Transfusion Practice in Hematological Patients of a Tertiary Care Center

Hematological patients were treated at La Fe University Hospital, which is a 1000-bedded tertiary care Center located in Valencia, Spain. Around 100 allogeneic and 60 autologous hematopoietic stem cell transplants are performed at the hospital each year. Patients can receive transfusions as inpatients and outpatients. Around 65% of all platelets are transfused to hematological patients, of which 20% are transfused in the outpatient setting. This study was approved by the Ethics Committee of the hospital.

Platelet requests were made by hematologists and fellows in the care of patients. These requests are not usually reviewed, and the patients received PLT transfusions as demanded. Periodically, retrospective audits of the platelet transfusion practice in the inpatient and outpatient settings are carried out with the aim of knowing the rate of appropriate transfusions. Transfusion episodes were consecutively reviewed for 3-month periods. The hematology fellows were in charge of carrying them out. Data were retrospectively collected for each transfusion episode for a patient. Information related to the indication for transfusion, the patient’s pre-platelet count, and the number of platelet products transfused was collected from the electronic clinical chart and from blood bank software (e-delphyn v.10.101.0.2, GPI Iberia, Madrid, Spain). Each transfusion event was defined as appropriate if adhering to guidelines or not appropriate in case of non-adherence. Audit standards were developed by the Spanish Society of Blood Transfusion (SETS), based on international guidelines, and were as follows: (a) The platelet count threshold for prophylactic transfusion was <10,000/µL. If fever, sepsis, coagulopathy, or acute promyelocytic leukemia were present, the threshold should be increased until the pre-transfusion platelet count is <20,000/µL. (b) The threshold for control of bleeding or increasing the platelet count for invasive procedures was < 50,000/µL. The dose of platelets to be transfused consisted of 4 units of pooled random-donor platelets or 1 unit of a single-donor platelet.

In addition, an audit on platelet transfusion threshold in different procedures was performed. The consecutive hematological patients who underwent lumbar puncture, bronchoscopy, and bone marrow aspirate, or trephine biopsy, were reviewed. Data from around 100 patients who underwent each procedure in 2022 were collected. Appropriateness rate was not analyzed.

### 2.2. Intervention: Development of Specific Platelet Transfusion Guidelines for Hematological Patients

We developed a specific platelet transfusion guideline for hematological patients at our hospital. The indications were based on previous recommendations, mainly from British and American Scientific societies (Table 1) [5,14]. There were no relevant differences as compared to the hospital’s available guide in the main recommendations for prophylactic and treatment intentions. Platelet thresholds for different procedures were not present in the previous guide but were included in the new guideline. The guide was delivered to the hematologists, who were encouraged to provide suggestions and comments. After 1 month, guidelines were discussed in a meeting and finally agreed upon with clinicians. The main controversial issue was the platelet threshold for outpatient transfusion and whether or not patients with stable chronic thrombocytopenia without active treatment should receive prophylactic transfusion. A year later, the transfusion burden was always analyzed, and a second audit was performed. Consecutive transfusion episodes were reviewed for a 4-month period.

### 2.3. Monitoring the Impact of Intervention Follow-Up

The guidelines were available in January 2024. We present data on the impact of the intervention over a period of 1 year (2024). Platelet transfusion activity was audited in mid-2024 and mid-2025, and these data were compared to the previous audits performed before the implementation of the guideline. We also reviewed the platelet transfusion activity and adverse effects related to from 2021 to 2024.

Every year, a training session on platelet transfusion practice is scheduled for hematologists and fellows in order to review and update the PLT transfusion indications.

### 2.4. Statistical Analyses

Computer software SPSS (version 15, SPSS Inc., Chicago, IL, USA) was used to perform the statistical analysis. Descriptive statistics are presented for variables. Results are expressed as median and range for continuous variables and as numbers with percentages for categorical variables. Qualitative variables were compared using the chi-square test or Fisher’s exact test. The Mann–Whitney U test for continuous variables was used to compare the groups when applicable. A *p* < 0.05 was considered significant.

## 3. Results

### 3.1. Platelet Transfusion Audit in Inpatient and Outpatient Settings

Results of audits 1 and 2 (before and post-intervention, respectively) are shown in the tables below. Table 2 shows the data of the audit performed in hospitalized patients, while Table 3 shows the results in outpatients. Audit 1 reviewed 128 inpatients and 49 outpatients transfused from March 2022 to September 2023. Audit 2 studied 130 inpatients and 45 outpatients requiring platelet transfusion from March 2025 to July 2025. All audits were retrospective. Results are expressed as median, minimum, and maximum values. Most patients received one platelet concentrate, while only six patients received two.

### 3.2. Platelet Transfusion Audit in Specific Procedures

The Table 4 below shows the results of an audit performed on hematological patients who received PLT transfusions before an invasive procedure. Results are shown as median, minimum, and maximum values (Table 4).

### 3.3. Platelet Transfusion Activity

**Figure 1:**
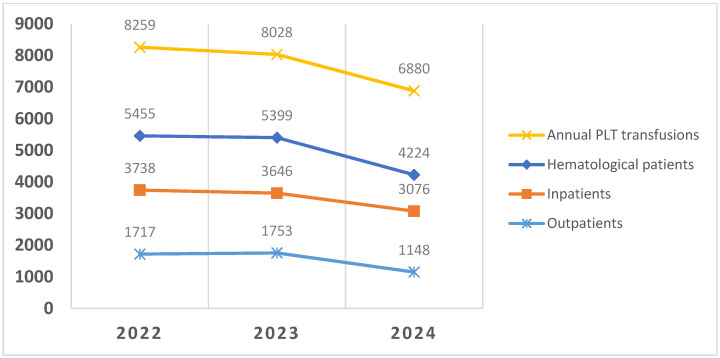
Platelet transfusions in hematological patients. Results are expressed as absolute numbers of platelet transfusion episodes per year.

Annual platelet transfusions decreased from 8028 before intervention to 6880 in 2024 (14% decrease). In the hematological area, where the intervention has been made, the reduction in platelet transfusions was 15.6% in hospitalized patients and 34.5% in outpatients as compared to 2023. The platelet supply decreased from 8407 in 2023 to 7138 in 2024 (*p* < 0.001). Adverse events related to PLT transfusion occurred in 51 patients in 2023 (0.63%) and 35 patients in 2024 (0.5%). Figure 1 shows the evolution of overall platelet transfusion activity in our hospital over the course of 3 years, and also in hematological inpatients and outpatients (*p* < 0.001 when comparing 2023 and 2024). Hematological inpatients and outpatients treated at the hospital were 25,915 and 19,931 in 2023, respectively, and 26,703 and 21,958 in 2024, respectively (*p* < 0.001).

## 4. Discussion

The intervention carried out in our department has had an impact on reducing platelet transfusion burden and promoting a more rational and standardized use of platelet transfusions, both in the inpatient and outpatient settings. The fact of establishing and spreading a platelet transfusion guide among hematologists is a feasible approach to implement a patient blood management strategy in hematological patients. Other approaches have been proposed as the introduction of the role of a platelet coordinator in the transfusion service to coordinate the platelet requests, the use of electronic clinical decision support systems [13], or the training of prescribers [10]. Unfortunately, our center does not have enough staff to dedicate a professional solely to this task, nor electronic systems to aid in transfusion decision-making, either. However, we are aware that any of these interventions would contribute to improving the transfusion practice.

Results of the first audits performed in hematological patients of our hospital showed us that the highest grade of inappropriate transfusions occurred in the prophylactic intention, both in inpatient (45%) and outpatient (54%) settings, and that inpatients were the most consumers of prophylactic transfusions. These results are quite similar to previous reported audits in centers similar to ours [9,15]. Then, the efforts to improve platelet transfusion practice must be focused on prophylactic intention. There was a general transfusion guideline available on our hospital website, but we considered that a new guide focused on hematological patients was needed and could better help physicians to make decisions on platelet transfusion practice. The hospital guide was more difficult to access, while the new guide could be found along with the clinical hematology documentation. The new guideline was quite similar to the previous one, based on international guidelines and gathering scientific evidence. After implementing the guide, the appropriate prophylactic transfusions in hospitalized patients have significantly increased from 55% to 75.6%. This improvement has also occurred, although to a lesser extent, in the outpatient subset. However, we consider the overall reduction in the platelet transfusion burden as a surrogate marker of improvement in platelet transfusion practice, since in many cases the most appropriate platelet is the one that is not transfused. Inpatient and outpatient activity was quite similar, and even higher in 2024 as compared to previous years.

On the other hand, there is little information about platelet transfusion practice in the outpatient setting. In fact, most available guidelines do not distinguish between inpatient and outpatient transfusions. O’Brien et al. reported a national survey of outpatient platelet transfusion practice in the USA [16]. These authors provided remarkable data as half of the respondents established specific outpatient platelet transfusion guidelines at their institution, and 51% used a threshold of less than 10,000/μL when transfusing stable, afebrile outpatients. Other thresholds used were 20,000/μL, 30,000/μL, and 40,000/μL. After discussing with clinicians, we established the threshold of 10,000/μL to transfuse outpatients, including those with chronic thrombocytopenia without active treatment or expected recovery. This agreement was challenging since hematologists are especially concerned about the risk of bleeding in severe thrombocytopenic outpatients. The common thought is that the more platelets are transfused, the lower the risk of bleeding. Based on this premise, without evidence and assuming that bleeding can always be prevented, transfusion is indicated on many occasions. Randomized studies showed that the appropriate use of platelet transfusion in hypoproliferative thrombocytopaenia reduces bleeding risk [17,18]. On the contrary, there is a lack of evidence supporting platelet transfusion in many other clinical scenarios. Therefore, physicians should take into account that severe thrombocytopenic patients have a high bleeding risk that cannot always be reduced or avoided even with platelet transfusion. The short half-life of platelets transfused (<4 days) should also be considered when considering a transfusion. Despite these concerns, greater adoption of a 10,000/µL threshold in the outpatient setting produced a 34.5% decrease in outpatient platelet transfusion burden in our center. However, it should be noted that a significant percentage of transfusions still fail to comply with the guidelines, so many patients still receive unnecessary transfusions and are put at risk of transfusion-related adverse effects. Therefore, a high potential for improvement remains.

Our results show the high variability in platelet transfusion practice among physicians, especially in the pre-procedure indications. In patients with similar platelet counts and similar clinical conditions and procedures, the indication of transfusion and the dose to be transfused or not is highly dependent on the physician. Again, the lack of evidence supporting the thresholds for different procedures and the different thresholds established in the available guidelines can explain this erratic practice [1,6,7]. Recently, the AABB has published platelet transfusion guidelines in which the platelet threshold for lumbar puncture is lowered to <20,000/µL [6]. This recommendation is based on the review of more than 1000 lumbar punctures performed with platelet counts < 50,000/µL. Spinal hematoma rates were as low as 0.8%. Then, guidelines on platelet transfusion are dynamic and change as more evidence is acquired. In fact, we have just updated our guidelines to include this recommendation.

We have also established thresholds for the most frequent procedures, but we have not performed an audit to assess the degree of compliance. This is a limitation of the present study.

In general, and as shown in our work and previous studies, physician adherence to platelet guidelines is poor [8]. Why clinicians do not follow the guidelines is complicated and difficult to assess. A lack of knowledge of current evidence and guidelines is one of the causes, since training improves transfusion practice [10,19]. In this line, ongoing training programs must be scheduled to educate medical staff and maintain good compliance with standards in the long term. Some actions that have been recommended to improve adherence are the dissemination of transfusion guidelines, revision of electronic requisition forms, and better recordkeeping [8].

As can be deduced from the previous paragraphs, many unknown issues remain in platelet transfusion practice that partly explain the variability among professionals: thresholds before different procedures according to the hemorrhagic risk, non-availability of other approaches to prevent bleeding, and lack of research on other parameters than platelet count to indicate the transfusion [20].

In conclusion, implementing PBM strategies focused on platelets contributed to a more rational and standardized use of platelet transfusions and decreased transfusion burden. Each organization should establish thresholds for platelet transfusion based on available evidence and guidelines, and also audit adherence to guidelines. Prescribing doctors must know that an unnecessary platelet transfusion (according to established hospital guidelines) puts the patient at risk and adds cost to the health system. In view of the scarce available evidence, research on platelet transfusion practice is mandatory.

## Figures and Tables

**Table 1:** Platelet thresholds established in the guideline for hematological patients.

Clinical Situation (Inpatients and Outpatients)	Platelet Threshold
Prophylactic use	<10,000/µL
Prophylactic use with additional risk factors (fever, sepsis, coagulopathy)	<20,000/µL
Acute promyelocytic leukemia	<30,000/µl
Bleeding	<50,000/µL
Multiple trauma or CNS injury/bleeding	<100,000/µL
**Pre-procedure**	
Major surgery	<50,000/µL
Bronchoscopy/liver biopsy	<50,000/µL
Lumbar puncture	<40,000/µL
Insertion of central venous catheter	<50,000/µL
insertion/removal of epidural catheter	<80,000/µL

**Table 2:** Audit on platelet transfusion in hospitalized hematological patients.

	Audit 1 (2022–2023)	Audit (2024–2025)	*p*
Patients (n)	128	130	
Platelet transfusion episodes (n)	288	315	
Prophylactic transfusions (n) Pre-transfusion count Appropriate (%)	245 11,000 (1000–47,000) 135 (55%)	279 9000 (1000–74,000) 211 (75.6%)	0.151 <0.001
Pre-procedure transfusions (n) Pre-transfusion count/µL Appropriate (%)	12 24,000 (15,000–45,000) 7 (58.3%)	33 21,000 (3000–67,000) 33 (100%)	0.222 0.006
Therapeutic transfusions Pre-transfusion count/µL Appropriate (%)	25 27,000 (2000–52,000) 22 (88%)	42 26,500 (4000–104,000) 36 (85.7%)	0.810 0.564

**Table 3:** Audit on platelet transfusion in hematological outpatients.

	Audit 1 (2022–2023)	Audit 2 (2024–2025)	*p*
Patients (n)	49	45	
Platelet transfusion episodes (n)	164	148	
Prophylactic transfusions (n) Pre-transfusion count/µL Appropriate (%)	154 11,000 (2000–35,000) 71 (46%)	125 10,000 (2000–160,000) 69 (54.3%)	0.396 0.332
Pre-procedure transfusions (n) Pre-transfusion count/µL Appropriate (%)	8 (1, 2, 4 and 5) 19,000 (7000–40,000) 2 (25%)	4 (1, 2, 3) 52,000 (13,000–65,000) 2 (50%)	0.109 0.648
Therapeutic transfusions Pre-transfusion count Appropriate (%)	2 25,000 (25,000–29,000) 2 (100%)	19 16,000 (3000–55,000) 10 (52.6%)	0.218 0.293

**Table 4:** Audit on platelet transfusion in hematological patients who underwent different procedures.

	Bone Marrow Aspirate or Trephine Biopsy	Lumbar Puncture	Bronchoscopy
Patients reviewed	123	98	107
Patients transfused (n) Platelet count/µL	17 13,000 (4000–42,000)	47 34,000 (8000–105,000)	62 39,000 (3000–88,000)
Transfused 1 PC * (n) Platelet count/µL	All	26 74,000 (21,000–105,000)	31 41,000 (10,000–88,000)
Transfused 2 PC (n) Platelet count/µl	0	21 34,000 (8000–105,000)	31 36,000 (3000–49,000)

* Platelet concentrate (apheresis or pooled).

## Data Availability

Data is contained within the article. Dataset available on request from the authors.

## Abbreviations

The following abbreviations are used in this manuscript:

PBM	Patient blood management
PLT	Platelets
